# Systematics and plastid genome evolution of the cryptically photosynthetic parasitic plant genus *Cuscuta *(Convolvulaceae)

**DOI:** 10.1186/1741-7007-5-55

**Published:** 2007-12-13

**Authors:** Joel R McNeal, Kathiravetpilla Arumugunathan, Jennifer V Kuehl, Jeffrey L Boore, Claude W dePamphilis

**Affiliations:** 1 Department of Plant Biology, University of Georgia, Athens, GA 30602, USA; 2 Department of Biology, Huck Institutes of the Life Sciences, and Institute of Molecular Evolutionary Genetics, The Pennsylvania State University, University Park, PA 16802-5301, USA; 3 Benaroya Research Institute at Virginia Mason, 1201 Ninth Avenue, Seattle, WA 98101, USA; 4 DOE Joint Genome Institute and Lawrence Berkeley National Laboratory, Walnut Creek, CA 94598, USA; 5 Genome Project Solutions, Hercules, CA 94547, USA

## Abstract

**Background:**

The genus *Cuscuta *L. (Convolvulaceae), commonly known as dodders, are epiphytic vines that invade the stems of their host with haustorial feeding structures at the points of contact. Although they lack expanded leaves, some species are noticeably chlorophyllous, especially as seedlings and in maturing fruits. Some species are reported as crop pests of worldwide distribution, whereas others are extremely rare and have local distributions and apparent niche specificity. A strong phylogenetic framework for this large genus is essential to understand the interesting ecological, morphological and molecular phenomena that occur within these parasites in an evolutionary context.

**Results:**

Here we present a well-supported phylogeny of *Cuscuta *using sequences of the nuclear ribosomal internal transcribed spacer and plastid *rps2*, *rbcL *and *matK *from representatives across most of the taxonomic diversity of the genus. We use the phylogeny to interpret morphological and plastid genome evolution within the genus. At least three currently recognized taxonomic sections are not monophyletic and subgenus *Cuscuta *is unequivocally paraphyletic. Plastid genes are extremely variable with regards to evolutionary constraint, with *rbcL *exhibiting even higher levels of purifying selection in *Cuscuta *than photosynthetic relatives. Nuclear genome size is highly variable within *Cuscuta*, particularly within subgenus *Grammica*, and in some cases may indicate the existence of cryptic species in this large clade of morphologically similar species.

**Conclusion:**

Some morphological characters traditionally used to define major taxonomic splits within *Cuscuta *are homoplastic and are of limited use in defining true evolutionary groups. Chloroplast genome evolution seems to have evolved in a punctuated fashion, with episodes of loss involving suites of genes or tRNAs followed by stabilization of gene content in major clades. Nearly all species of *Cuscuta *retain some photosynthetic ability, most likely for nutrient apportionment to their seeds, while complete loss of photosynthesis and possible loss of the entire chloroplast genome is limited to a single small clade of outcrossing species found primarily in western South America.

## Background

**Figure 1:**
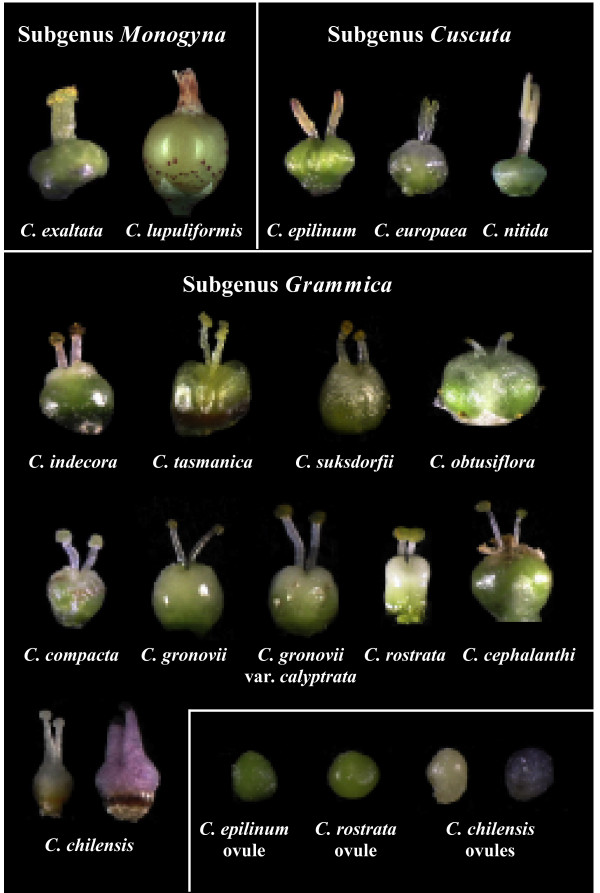
**Gynoecia and ovules of species across the taxonomic diversity of *Cuscuta***. Species in subgenus *Monogyna *have fused styles, species in subgenus *Cuscuta *have linear stigmas and species in subgenus *Grammica *have globose stigmas. All species examined had chlorophyllous ovules and gynoecia except *C. chilensis*, for which two different flower morphs with gynoecia of various shapes, sizes and colors were examined.

Between 150 and 200 species of *Cuscuta *have been described, and they are distributed widely on every continent except Antarctica [1]. These parasites have no roots at maturity and their leaves are reduced to minute scales. As such, few morphological characters exist to distinguish and classify species outside of the flower and fruit. Style and stigma morphology, capsule dehiscence and corolla and calyx shape and size form the basis of existing monographical studies [1-3]. Engelmann [2] separated *Cuscuta *into three subgenera on the basis of style fusion and stigma shape. Members of subgenus *Monogyna *have the two styles fused for most or all of their length, and consist of thick-stemmed species that commonly parasitize trees and shrubs; subgenera *Cuscuta *and *Grammica *have free styles, with stigmas being globose in subgenus *Grammica *and elongate in subgenus *Cuscuta *(Figure 1). The last full monograph of the genus completed by Yuncker [1] recognized nine species in *Monogyna*, distributed primarily in Eurasia and Africa with one species, *Cuscuta exaltata *Engelmann, having a disjunctive distribution in the southern United States in the scrub habitat of Florida and Texas. The 28 species in subgenus *Cuscuta *recognized by Yuncker have native ranges restricted to, but widely distributed in, the Old World. Subgenus *Grammica*, with 121 species recognized by Yuncker, is almost completely limited to the New World, with a handful of exceptions in Asia, Africa and the Pacific islands, including Tasmania and Australia.

Engelmann [2] further divided each of the subgenera into sections based on stigma morphology and capsule dehiscence. *Monogyna *consists of two sections; the first, Callianche, contains only *Cuscuta reflexa *Roxburgh, defined by its elongated stigmas atop the fused styles. All other members of subgenus *Monogyna *are relegated to section Monogynella, which have shorter, stouter stigmas. All members of subgenus *Monogyna *possess a circumscissile capsule as the fruit. Subgenus *Cuscuta *is subdivided into four sections. Section Cleistococca has only one species, *Cuscuta capitata *Roxburgh, which is distinguished from all other members of subgenus *Cuscuta *by having an indehiscent capsule as its fruit. Fruits of sections Pachystigma and Epistigma are only irregularly circumscissile, and fruits of section Eucuscuta are always cleanly dehiscent. Section Pachystigma is distinguished from section Epistigma by the presence of long, slender styles topped by wider stigmas, whereas members of section Epistigma possess only short to undetectable styles topped by the elongated stigmas. The six species of Pachystigma are restricted to Southern Africa, while the four species of Epistigma and *Cuscuta capitata *are restricted to central Asia. Section Eucuscuta has a wider distribution, with the largest number of species found close to the Mediterranean Sea. Subgenus *Grammica *is divided into two sections based on capsule dehiscence, with section Eugrammica possessing complete to partially dehiscent capsules and section Cleistogrammica producing indehiscent capsules. Species of subgenus *Grammica *are relatively evenly divided between the two subgenera, with 53 species in section Cleistogrammica and 68 species in Eugrammica [1].

*Cuscuta *is a readily recognizable genus, with the only species in the completely unrelated but strikingly similar parasitic vine genus *Cassytha *L. (Lauraceae) ever likely to cause any confusion [4] ; however, small flowers and a paucity of usable morphological characteristics often make the identification of *Cuscuta *to the species level a challenge. Although no comprehensive taxonomic study of the entire genus has been completed since Yuncker's monograph, *Cuscuta *remains one of the most widely studied parasitic plant lineages, with numerous publications on its anatomy [5-7], nutritional physiology [8] , plastid evolution [9-21] and even foraging behavior [22-25]. Phylogenies of Convolvulaceae with a small sampling of *Cuscuta *species showed it is confidently nested within that family [26]. Although its exact placement could not be strongly inferred with more in-depth analysis [27] , the most confident placement was sister to a the 'Convolvuloideae' clade [28]. Taxa from subgenus *Monogyna *appeared basal to subgenus *Cuscuta *and subgenus *Grammica *in those studies. Another study showed multiple members of subgenus *Cuscuta *to be nested within multiple clades of subgenus *Grammica *[21] , although those data are likely a result of misidentification of taxa and are discussed more extensively in our results.

Conflicting evidence exists as to the photosynthetic ability across the genus. Machado and Zetsche [9] demonstrated low levels of photosynthetic carbon assimilation in the noticeably chlorophyllous stems of *Cuscuta reflexa *(subgenus *Monogyna*) despite apparent loss of all *ndh *genes [12] , but found no detectable levels of RuBisCo expression in *C. europaea*, despite the presence of the gene encoding its large subunit (*rbcL*) in the plastid genome. Studies further showed that *C. reflexa *only produces chlorophyll in a specific layer of cells isolated from atmospheric gas exchange, suggesting it only photosynthesizes by recycling carbon dioxide released from respiratory byproducts of carbohydrates from its host source [5]. *C*. *pentagona *Engelmann of subgenus *Grammica *was shown to possess a normal photosynthetic ratio of chlorophyll a to b, contain properly localized photosynthetic proteins and display low levels of carbon assimilation [29]. However, other members of subgenus *Grammica *seem to possess highly altered plastid genomes; *C. gronovii *Willdenow and *C. subinclusa *Durand et Hilgard seemingly lack plastid-encoded polymerase (*rpo*) genes [18] , although low levels of transcription of *rbcL *still take place from nuclear-encoded polymerase promoter sites [19] , and these species, along with *C. campestris *Yuncker and *C. reflexa *still possess normal chlorophyll a and b ratios [16]. In contrast, *C. odorata *Ruiz et Pavon and *C. grandiflora *Humbolt, Bonpland et Kunth are achlorophyllous, lack thylakoids and do not produce detectable levels of *rbcL *transcript or protein [16]. The additional loss of some non-coding data from the plastid genome along with a few minor changes to intact reading frames within *Cuscuta *and Convolvulaceae have been reported and roughly mapped on a phylogeny of *Cuscuta *based on a minimal sampling of taxa [20].

In this study, we examine the phylogeny of the genus *Cuscuta *by sampling 35 species from all sections of the genus defined by Englemann [2] with the exceptions of section Epistigma and the monospecific section Cleistococca. Our sampling also includes species from 19 of 29 subsectional groups recognized by Yuncker [1]. We obtain DNA sequences for phylogenetic analysis from two plastid loci (*rbcL *and *rps2*) and the nuclear internal transcribed spacer (ITS) region between the 18S and 5.8S ribosomal RNA loci from largely overlapping subsets of taxa to investigate phylogenetic relationships within the genus and test the monophyly of the previously defined subgeneric and subsectional delimitations. We determine genome sizes for species available as fresh tissue in order to address questions of species delimitation and to test whether genome size correlates with published chromosome numbers, which are highly variable [30]. In addition to the plastid loci mentioned above, which correspond to the RuBisCo large subunit and a small ribosomal protein subunit respectively, we sample two more plastid loci representing two other functionally distinct genes (*atpE*, ATP synthase subunit; *rpoA*, plastid-encoded polymerase subunit) from smaller subsets of taxa in order to test whether all classes of plastid genes are evolving equally in *Cuscuta *relative to photosynthetic taxa. Using further polymerase chain reaction (PCR) assays, we test the distribution of major changes to the plastid genome within the genus and combine them with previously published evidence to gain a comprehensive view of photosynthetic evolution within *Cuscuta*. Finally, we use evidence from the biology and natural history of these parasites to suggest potential hypotheses as to why photosynthesis is retained in most members of the genus despite what superficially appears to be minimal opportunity for gain of photosynthetic carbohydrate.

## Results

### Phylogeny

**Figure 2:**
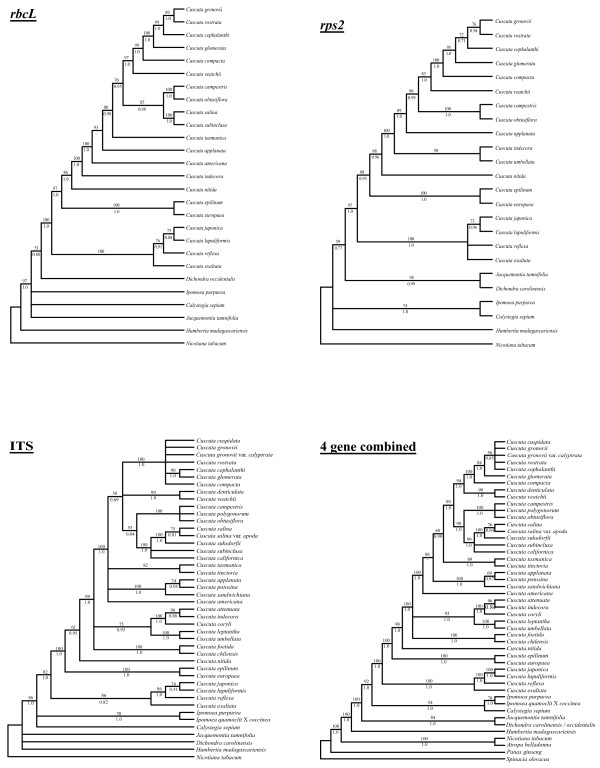
**Maximum parsimony bootstrap consensus trees**. Consensus trees of 500 bootstrap replicates for plastid *rbcL*, plastid *rps2*, nuclear ITS and all three genes combined with plastid *matK*. Parsimony bootstrap values are shown above the branches at nodes above 50% support, while Bayesian posterior probabilities are given below the branches.

**Figure 3:**
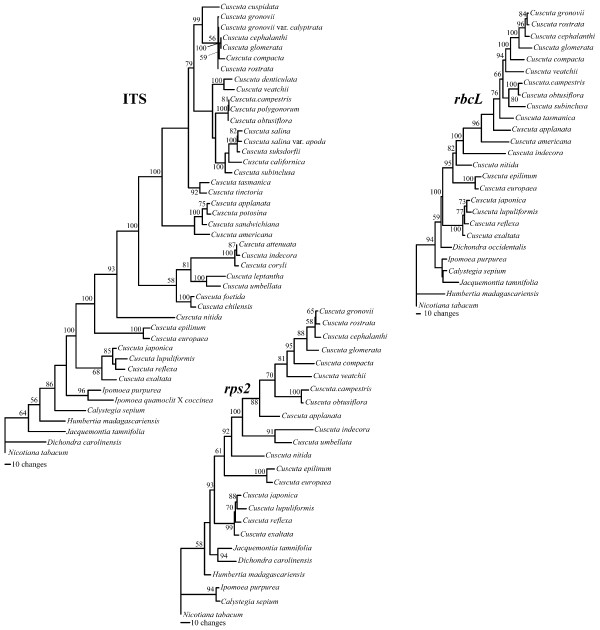
**Maximum-likelihood phylograms**. Phylograms of individual genes produced by maximum likelihood with bootstrap values shown at the nodes.

**Figure 4:**
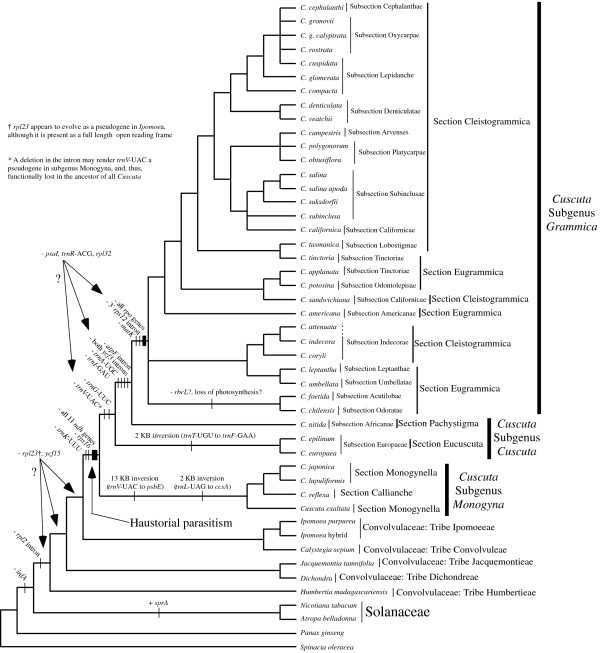
**Cladogram mapping taxonomic classifications and plastid genome changes onto recovered phylogeny**. Parsimony bootstrap consensus tree (500 replicates) with taxonomic classifications according to Yuncker [1] to the right of taxon names. Changes to the plastid genome inferred via parsimony mapping are shown on the nodes.

Figure 2 shows individual parsimony bootstrap consensus cladograms for ITS, *rps2 *and *rbcL *and the four-gene combined dataset including *matK *data. Maximum parsimony bootstrap values (MP) are shown above the nodes and Bayesian posterior probability estimates (PP) are shown below the nodes. The individual gene trees are almost identical in topology, with no well-supported incongruences. Many of the support values are high for individual genes and almost every node is very well supported in the combined analysis. Furthermore, maximum-likelihood analyses were performed on the individual gene datasets; these analyses also gave nearly congruent topologies that agreed at well-supported in-group nodes (Figure 3). *Cuscuta *was found to be sister to the 'Convolvuloideae' clade [28] for two of the genes (*matK *and ITS), and this placement was very well supported in the combined analysis (MP 92, PP 1.0). Within *Cuscuta*, subgenus *Monogyna *was monophyletic and sister to all other *Cuscuta *species (MP 100, PP 1.0), with *C. exaltata *sister to all other sampled *Monogyna *species. Section Monogynella was paraphyletic, with *C. reflexa *of the monotypic section Callianche nested within (MP BP 100, PP 1.0). Subgenus *Cuscuta *was strongly supported as paraphyletic (MP 98, PP 1.0), with *Cuscuta nitida *Meyer representing section Pachystigma falling sister to subgenus *Grammica*, a result also supported by loss of two transfer RNA genes and loss of introns from *ycf3 *and *atpF *(see Figure 4). The two sampled species in section Eucuscuta were monophyletic (MP 100, PP 1.0). Subgenus *Grammica *was clearly monophyletic (MP 100, PP 1.0), although many highly supported nodes reject the monophyly of sections Eugrammica and Cleistogrammica. The basal lineage of subgenus *Grammica *was not clearly resolved, with the consensus showing a clade including subsection Odoratae (*C. chilensis *Ker-Gawler) with subsection Acutilobae (*C. foetida *Humboldt, Bonpland et Kunth) and a clade with subsections Indecorae, Umbellatae and Leptanthae in a polytomy together with a clade containing the remainder of the sampled subsections of subgenus *Grammica*. Subsection Californicae and subsection Tinctoriae were not monophyletic in the combined four-gene tree, but the monophyly of all other subsections cannot be disputed by these data. Our data are congruent at well-supported nodes with a study that sampled many additional species of subgenus *Grammica *utilizing two short loci (including ITS) [31].

### Nuclear genome size results

Genome size estimates were highly variable within *Cuscuta *and did not appear to be related to previously published chromosome numbers overall (Table 1). Species in subgenus *Monogyna*, which generally show intermediate chromosome numbers between the other two subgenera [30] , have extremely large nuclear genomes according to our results. Low numbers of plastid clones relative to nuclear clones in a genomic fosmid library used to generate the full plastid genome sequence of *Cuscuta exaltata *help confirm these data [32]. Within subgenus *Cuscuta *section Eucuscuta, genome sizes of *Cuscuta europaea *L. and *C. epilinum *Weihe actually did appear to correlate with karyotypes and known ploidy levels [30] , with the apparent recent triploid *C. epilinum *having a genome size consistent with these data relative to *C. europaea*. Estimated nuclear genome sizes within subgenus *Grammica *are the most variable, with an estimate for *Cuscuta pentagona *(1.16 picograms/2C) being the smallest of all sampled species and *C. indecora *Choisy (65.54 pg/2C) being the largest. There does not appear to be a standard genome size within this subgenus, although closely related species in subsection Oxycarpae, subsection Cephalanthae and subsection Lepidanche all possess proportional nuclear genome size, with three size classes perhaps reflecting different ploidy levels. Interestingly, accessions of *C. gronovii *from different geographic localities showed quite striking differences in genome size, even within two collections made within the state of Pennsylvania. Smaller, secondary peaks were detected in many species, suggesting that these stem tips were growing so rapidly as to have many cells at different stages of mitosis with different overall DNA content depending on phase. Alternatively, the parasites could be undergoing endoreduplication, a process frequent in metabolically active cells of eukaryotes by which the genome of those cells is doubled within the nucleus [33].

**Table 1:** Genome size and chromosome numbers in *Cuscuta*

	Species	Nuclear genome size estimate (pg/2C)	SD	Published chromosome estimate (2*n*)
Convolvulaceae				
	*Ipomoea purpurea*	1.51	0.020	30
				
Subgenus *Monogyna*				
	*Cuscuta exaltata*	41.86	0.559	?
	*Cuscuta lupuliformis*	44.93	0.290	28
				
Subgenus *Cuscuta*				
	*Cuscuta epilinum*	7.74	0.177	42
	*Cuscuta europaea*	2.15	0.046	14
				
Subgenus *Grammica*				
	*Cuscuta chilensis*	5.73	0.074	? (*C. odorata *= 32)
	*Cuscuta indecora*	65.54	0.572	30
	*Cuscuta obtusiflora*	1.58	0.022	?
	*Cuscuta polygonorum*	1.62	0.018	?
	*Cuscuta campestris*	10.83	0.290	56
	*Cuscuta pentagona*	1.16	0.023	56,44
	*Cuscuta veatchii*	5.83	0.096	? (*C. denticulata *= 30)
	*Cuscuta compacta*	15.69	0.056	30
	*Cuscuta rostrata*	8.12	0.015	?
	*Cuscuta cephalanthi*	7.85	0.029	60
	*Cuscuta gronovii *(NJ)	7.56	0.129	60
	*Cuscuta gronovii *(OH)	7.17	0.109	...
	*Cuscuta gronovii *(C PA)	13.81	0.074	...
	*Cuscuta gronovii *(SE PA)	4.37	0.194	...
	*Cuscuta gronovii calyptrata*	11.47	0.130	...

Geographic locations are given in parentheses for different accessions of *Cuscuta gronovii*. Although genome size data was unavailable for *Cuscuta odorata *and *C. denticulata*, they are close relatives of *C. chilensis *and *C. denticulata *respectively and so their published chromosome counts are listed. Standard deviations for all genome size estimates are based on four measurements.

### Plastid genome variation assays

**Figure 5:**
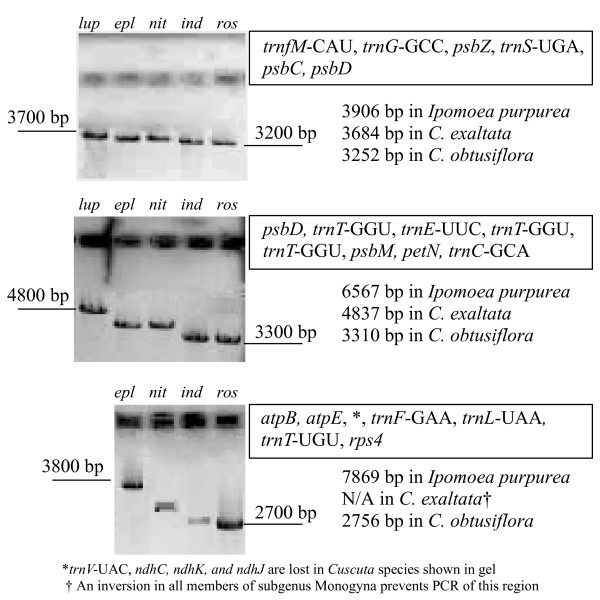
**Results of long PCR tests to detect differences in intergenic spacer regions**. Here *trnfM*-CAU to *psbD *(top), *psbD *to *trnC*-GCA (middle) and *atpB *to *rps4 *(bottom) are shown. Lengths are calculated from complete plastid genome sequences of *Ipomoea purpurea, Cuscuta exaltata *and *C. obtusiflora *are shown beneath genes contained within each region.

Major changes to the plastid genome reported in this and previous studies are mapped on the cladogram in Figure 4. PCR and sequencing of the region between *petD *and *rps11 *showed that taxa across subgenus *Grammica *contained only residual *rpoA *pseudogene sequence, although the length of the remaining intergenic region was surprisingly constant across those taxa (data not shown). This confirmed previous hybridization data that failed to detect *rpo *(plastid-encoded RNA polymerase) genes [18,20] and showed loss of transcription from known plastid-encoded polymerase promoter sites [18]. PCR data also detected an inversion in the large single-copy region of *C. reflexa *[11] and *C. japonica *[20] that is a synapomorphy in all sampled species of subgenus *Monogyna*, as is a constriction of the large single-copy boundary of the inverted repeat region into *ycf2*. A two-kilobase inversion in the large single-copy region of the plastid genome was found in both sampled members of subgenus *Cuscuta *subsection Eucuscuta. Long PCR covering many intergenic regions demonstrated that the substantial reduction of non-coding DNA is shared across subgenus *Grammica*, with all species in the subgenus seemingly converging on a minimal length (Figure 5). Sequences from *Cuscuta lupuliformis*, in subgenus *Monogyna*, show much less reduction in intergenic regions. Members of subgenus *Cuscuta*, which still possess a full set of seemingly functional *rpo *genes, show intermediate levels of intergenic sequence loss; this indicates that intergenic constriction does not completely result from a loss of plastid-encoded polymerase promoter regions.

Finally, we attempted to study plastid genes in *C. chilensis*. *C. chilensis *is an achlorophyllous relative of *C. odorata*, a species which appears to lack *rbcL *[16]. Unlike the results from *C. odorata*, we were unable to amplify *rrn16 *from *C. chilensis *using many combinations of primers. Furthermore, hybridization of various ribosomal protein gene and *rrn16 *PCR products from other species within *Cuscuta *subgenus *Grammica *to a filter containing over 1,500 *Cuscuta chilensis *clones from a genomic fosmid library returned no positive hits. Positive control amplifications of *Cuscuta chilensis *mitochondrial genes and hybridization of mitochondrial probes to the *Cuscuta chilensis *library showed that organellar DNA was present in our DNA extraction and library.

### Tests of selective constraint

**Figure 6:**
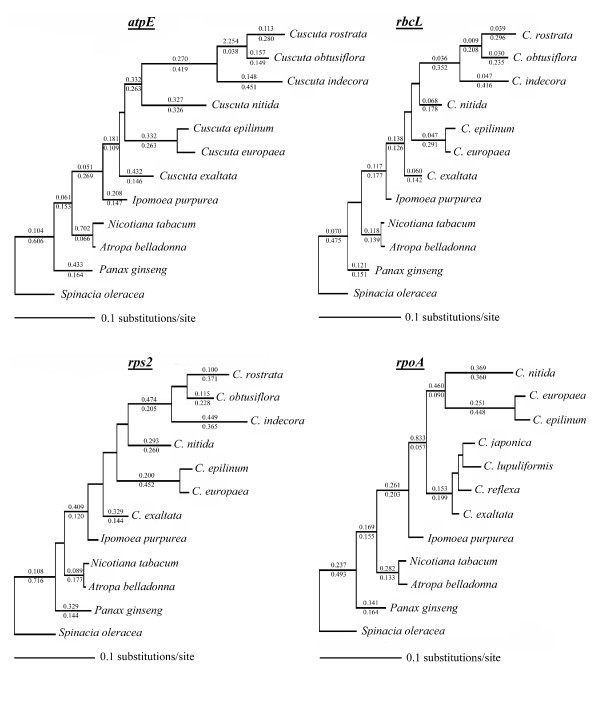
**Unconstrained maximum-likelihood tree estimates for *atpE*, *rbcL*, *rps2 *and *rpoA***. Non-synonymous to synonymous rate ratio values calculated in HyPhy are shown above and synonymous rate values are shown below all branches with overallrate values greater than 0.02.

With such variability in gene content across *Cuscuta*, it was important to determine whether remaining genes are still under selective constraint and how these patterns of constraint vary across genes, across the taxonomic range of *Cuscuta *and between *Cuscuta *and its photosynthetic relatives. Unconstrained maximum-likelihood trees are shown in Figure 6. Trees with all branches constrained to the same non-synonymous to synonymous rate ratio were significantly worse than fully unconstrained trees for *atpE*, *rbcL *and *rps2 *(Table 2), indicating lineage-specific heterogeneity in selective constraint for these genes. No significant difference was observed between the likelihoods of *rpoA *trees when trees with all branches constrained to an identical non-synonymous to synonymous rate ratio were compared with unconstrained trees. Of the four hypotheses tested for *atpE *(significant constraint differences between outgroups and all Convolvulaceae including *Cuscuta*, differences between *Cuscuta *and non-parasites, differences between subgenera *Cuscuta*+*Grammica *and all other taxa, and differences between subgenus *Grammica *and all other taxa), constraining an independent non-synonymous to synonymous rate ratio for all *Cuscuta *from the rest of the tree most improved the likelihood scores, with the resulting likelihood no longer being significantly different from the fully unconstrained tree. For *rbcL*, all of the clades tested in the same manner remained significantly worse than the unconstrained tree, with the greatest improvement coming when subgenera *Cuscuta *and *Grammica *together were given a separate non-synonymous to synonymous rate ratio. In this case, as is apparent in the unconstrained tree, the non-synonymous to synonymous rate ratio actually decreases within *Cuscuta*, with all species under higher levels of purifying selection than the autotrophic outgroups. For *rps2*, yet a third pattern was observed. Of the hypotheses tested, a change in non-synonymous to synonymous rate ratio across Convolvulaceae improves the likelihood the most, again to the extent that it is no longer significantly different to the unconstrained tree, suggesting that a relaxation of constraint may have occurred in this gene before the evolution of parasitism. A similar result was found in the independently derived parasitic plant family Orobanchaceae, where significant rate increases in *rps2 *are seen even in very photosynthetic lineages before evolution of holoparasitism [34]. For *rpoA*, there was no significant difference between the fully constrained and fully unconstrained trees, and no appreciable changes occurred under any of the proposed hypothetical shifts in non-synonymous to synonymous rate ratio.

**Table 2:** LRT comparisons of trees with constrained clades versus fully unconstrained trees

Constrained branches	*d*_*N*_*/d*_*S*_	*p*-value	Degrees of freedom
***atpE***			
All	0.256	0.040	20
			
Convolvulaceae	0.284		
All but Convolvulaceae	0.193	0.049	19
			
*Cuscuta*	0.323		
All but *Cuscuta*	0.168	0.120	19
			
Subgenus *Grammica *+ subg. *Cuscuta*	0.323		
All but subg. *Grammica *+ subg. *Cuscuta*	0.168	0.000	19
			
Subgenus *Grammica*	0.238		
All but subgenus *Grammica*	0.264	0.032	19
			
***rbcL***			
All	0.071	0.001	20
			
Convolvulaceae	0.057		
All but Convolvulaceae	0.111	0.006	19
			
*Cuscuta*	0.052		
All but *Cuscuta*	0.111	0.011	19
			
Subgenus *Grammica *+ subg. *Cuscuta*	0.047		
All but subg. *Grammica *+ subg. *Cuscuta*	0.108	0.047	19
			
Subgenus *Grammica*	0.094		
All but subgenus *Grammica*	0.046	0.011	19
			
***rps2***			
All	0.207	0.003	20
			
Convolvulaceae	0.265		
All but Convolvulaceae	0.098	0.127	19
			
*Cuscuta*	0.249		
All but *Cuscuta*	0.140	0.012	19
			
Subgenus *Grammica *+ subg. *Cuscuta*	0.249		
All but subg. *Grammica *+ subg. *Cuscuta*	0.165	0.005	19
			
Subgenus *Grammica*	0.238		
All but subgenus *Grammica*	0.192	0.002	19
			
***rpoA***			
All	0.322	0.247	20
			
Convolvulaceae	0.357		
All but Convolvulaceae	0.259	0.331	19
			
*Cuscuta*	0.360		
All but *Cuscuta*	0.276	0.296	19
			
Subgenus *Cuscuta*	0.333		
All but subg. *Cuscuta*	0.316	0.203	19
			
*Cuscuta nitida*	0.389		
All but *C. nitida*	0.313	0.223	19

## Discussion

### Morphological, biogeographical and taxonomic interpretation of phylogeny

**Figure 7:**
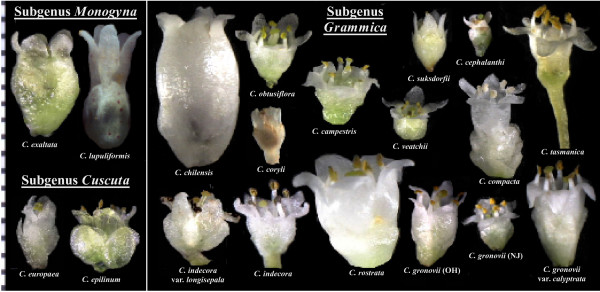
**Floral diversity within the genus *Cuscuta***. Ruler marks to the left are millimeters. *Cuscuta chilensis *and *C. rostrata *were unable to produce selfed seed when hand-pollinated, whereas all other species readily produced selfed seed with no assistance. *C. coryli *flower is rehydrated from an herbarium specimen; all other flowers were collected fresh from the Pennsylvania State University greenhouse.

Although subgenus *Grammica *is clearly monophyletic in our study, it has been suggested that it is paraphyletic, with members of subgenus *Cuscuta *nested in multiple clades within *Grammica *[21]. That study also included data from plastid *rbcL *and nuclear ITS, allowing us to compare sequences for taxa shared with our study. As their phylogenies show strong conflict with ours and make no sense from a morphological standpoint, and because data reportedly gathered from the same species as vouchered specimens from our study clearly represent unrelated taxa, we conclude that multiple taxa were misidentified in [21]. This likely also affected their conclusion that loss of photosynthetic genes is distributed randomly on the phylogeny, when a clear stepwise and more parsimonious loss of photosynthetic genes is evident from our results. *Cuscuta *species can be difficult to identify when in flower (see Figure 7) and nearly impossible to identify from vegetative material, which was the source of tissue used for DNA isolations [12].

**Figure 8:**
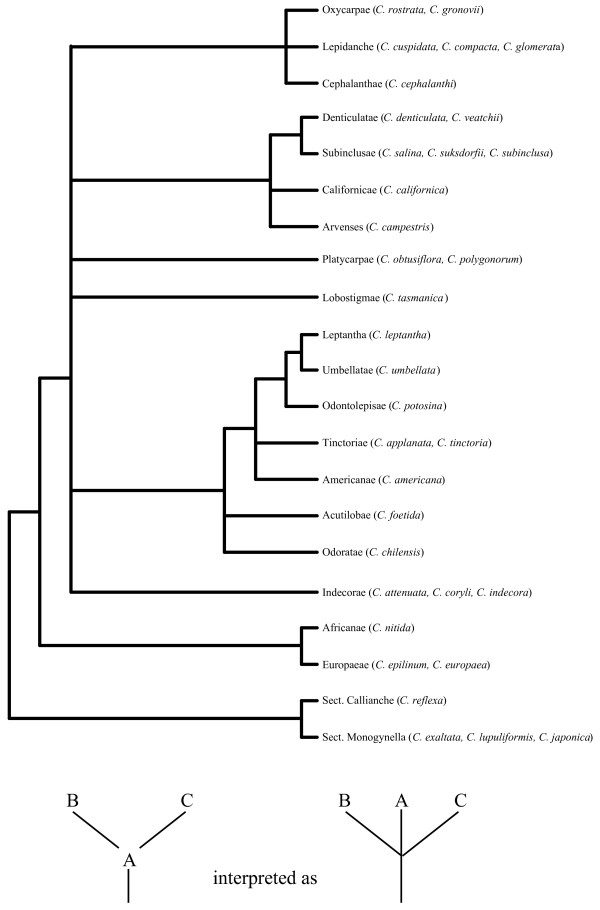
**Approximation of phylogenetic inferences suggested by Yunker**. A rough phylogeny by Yunker was provided in the most recent monograph of the entire *Cuscuta *genus on page116 of [1]. In cases where one or more subsection was shown by Yunker to arise from another, the given node in the tree was converted to a polytomy between the presumed progenitor subsection and its 'offspring' subsections. Taxa included in this study are shown to the right of subsection classifications to which they belong.

Yuncker believed that the morphological features of subgenus *Grammica *were the ancestral states owing to the species-richness of that subgenus; subgenus *Grammica *is clearly in a highly derived position within the genus and cannot be considered a potentially ancestral group. However, once the tree is re-rooted to the proper node (Figure 8), subsectional relationships within sections largely agree with interpretation of phylogenetic relationships proposed by Yuncker. Artificial relationships found to be non-monophyletic mostly result from interpretation of two morphological characters: stigma morphology and capsule dehiscence. Elongated stigmas appear to be a derived state in *C. reflexa*, which is nested within a clade of species with much stouter stigmas. In contrast, the globose stigmas seen in subgenus *Grammica *are apparently derived from elongate stigmas, such as those seen in subgenus *Cuscuta*. Stigma morphology appears to be quite plastic within the genus and a full range of intermediates between subgenus *Cuscuta *and subgenus *Grammica *exist. Thus, it is not surprising that section Pachystigma (represented by *C. nitida *in our dataset), with intermediate stigma morphology, is actually sister to subgenus *Grammica *and should be included in that subgenus. In fact, a species within section Pachystigma, *Cuscuta cucullata *Yuncker, is so similar to the only member of subgenus *Grammica *found in South Africa, *C. appendiculata *Engelmann, that Yuncker points out that they may be confused with each other. Although we were unable to sample those two species for our phylogeny, their distribution in South Africa has biogeographical implications for the colonization of the New World by subgenus *Grammica *from a South African/South American dispersal event. Putatively basal clades of subgenus *Grammica *are either distributed almost completely in South America (subsection Acutilobae and subsection Odoratae) or contain lineages distributed widely from South to North America (subsection Indecorae and subsection Umbellatae). Interestingly, *C. cucullata *and *C. appendiculata *are unique among South African *Cuscuta *species in having indehiscent capsules, which facilitate floating and water-mediated dispersal of the seeds in many members of subgenus *Grammica *section Cleistogrammica. Subgenus *Grammica *has successfully spread across both North and South America since colonizing the New World and now contains many more species than the other two subgenera combined. Whether the ancestor of *C. exaltata *(subgenus *Monogyna*) may have taken a similar route to colonize the New World is unknown, although it too shares a morphologically similar relative in South Africa (*C. cassytoides *Nees von Esenbeck).

While capsule dehiscence was one of the main characters used for monographical work in *Cuscuta *[1,2] , our phylogenetic analyses agree with another study [31] that it is a transient character in the genus with very little systematic value and that the sectional entities of Eugrammica and Cleistogrammica should no longer be recognized. Many species of *Cuscuta *subgenus *Grammica *possess irregularly dehiscent capsules that are not easily classified as either indehiscent or circumscissile. Two interesting cases of indehiscent-capsuled species being allied to clades with circumscissile capsules are *C. tasmanica *Engelmann and *C. sandwichiana *Choisy. These derived members of subgenus *Grammica *have independently colonized islands far from the home of their Mexican sister taxa and both are found in coastal habitats. Indehiscent capsules may have also aided their aquatic dispersal events. Other taxa from subgenus *Grammica *found in the Pacific Rim (e.g. *C. australis *R. Brown) likely took a similar dispersal route via indehiscent capsules [31] , although we do not have data for those taxa in our phylogeny. Two other Old World species from subgenus *Grammica*, *Cuscuta chinensis *in Asia and *Cuscuta kilamanjari *in Africa, have dehiscent capsules, and may or may not have dispersed to their present ranges via ancestral indehiscent capsules.

### Genome sizes and speciation

Estimates of species number within *Cuscuta *vary greatly, largely because so few characters exist to distinguish them. The existence of forms with supernumary chromosomes [35] and such widely scattered estimates of chromosome numbers in the genus [30] suggest polyploid and aneuploid evolution may occur rather rapidly in this lineage. Species that appear very similar morphologically may occupy very dissimilar ecological niches and exhibit different host preferences. One such example involves *C. pentagona*, *C. campestris*, *C. polygonorum *Engelmann and other relatives in subsection Arveses and subsection Platycarpae. *C. campestris *is often merged taxonomically with *C. pentagona*, as the two are distinguished primarily by slight differences in overall flower size and angularity of the calyx. However, our estimates of genome size between accessions identified as either form differed in size by almost a factor of 10 (Table 1). Estimates for *C. polygonorum *and *C. pentagona *differ by almost 50%, although those species have also been merged in at least one taxonomic treatment [36]. *C. polygonorum *can be identified by flowers that are often four-merous and that have a slightly different gynoecium shape than those in *C. pentagona*. However, the species can usually be distinguished simply by noticeable habitat and host preferences. In such cases, where forms seem to be ecologically distinct as well as morphologically distinguishable, we suggest species-level distinction is likely warranted given the disparate genome sizes. Seemingly different ploidy levels exist within *Cuscuta gronovii*. Morphological variation in corolla size and shape exist in this species as well (Figure 7), indicating that cryptic species with different chromosome numbers that are incapable of interbreeding may exist. Accelerated rates of nucleotide substitution in the nuclear genome may also promote rapid speciation in subgenus *Grammica *if acceleration in ribosomal loci such as ITS (Figure 3) and 18S [37] are correlated with protein-coding rates. As almost all species of *Cuscuta *readily produce selfed seed even in the absence of pollinators, and pollen is often deposited on the stigma before the corolla opens, drastic changes in the nuclear genome that prevent outcrossing may promote speciation.

### Plastid genome evolution in *Cuscuta*

In contrast to previous descriptions of chloroplast genome evolution in *Cuscuta *as a 'slippery slope' [20] or as occurring in a random, uncoordinated manner across the phylogeny [21] , we find that plastid genome evolution in *Cuscuta *has occurred in a stepwise fashion, with punctuated modification at various evolutionary time-points followed by long periods of stasis within various clades. Major changes occurred in the ancestor of the genus, the ancestor of subgenus *Grammica *and within one fully non-photosynthetic clade of subgenus *Grammica*. Across most species of subgenus *Grammica *and, as such, the majority of all *Cuscuta *species, plastid genome content appears to have stabilized on a smaller, but constrained size (see, e.g., Figure 5). Different types of genes appear to be evolving under different levels of constraint. Most surprisingly, *rbcL *appears to be under much greater purifying selection in *Cuscuta *than in autotrophic relatives. This effect may largely be a result of much higher overall rates of substitution in *Cuscuta *for the plastid genome (see branch lengths in Figures 3 and 6), but a need for amino acid stasis in *rbcL*. This intense conservation of most photosynthetic genes is quite unexpected for a genus that lacks leaves and extensive chlorophyllous surface area. Hibberd *et al*. [5] suggest that recycling of internally respired carbon dioxide may be the answer. However, loss of *ndh *genes could possibly make these parasites extremely susceptible to photorespiration unless extremely high respiratory rates existed near these photosynthetic cells or some other mechanism similar to C4 photosynthesis existed [38]. Furthermore, these plants have seemingly little need to produce carbohydrates, which are readily obtained from the host.

A second pathway involving *rbcL *in lipid biosynthesis in green seeds of *Brassica *[39] suggests a tantalizing explanation for retention of photosynthetic genes in *Cuscuta*. Chlorophyll is concentrated in the developing ovules of *Cuscuta *(Figure 1), almost exclusively so in healthy members of subgenera *Grammica *and *Cuscuta*. Seeds often have high lipid content as energy reserves for the seedling and to aid in desiccation tolerance and seed longevity, and *Cuscuta *has been shown to accumulate lipid bodies that fill the majority of the non-nuclear cytoplasm [7]. Most *Cuscuta *species are annuals and must be prolific producers of highly energetic seeds to ensure at least some offspring will be able to germinate and survive long enough to search out and attach to a host. The seeds are impermeable to water until the epidermal layer is scarified and they can live unimbibed for decades and remain viable. As lipids are less available from vascular extracts from the host and because of the intense demand for lipid production during fruiting, this efficient lipid synthesis pathway is a more plausible explanation for conservation of a photosynthetic apparatus in *Cuscuta *than residual carbohydrate production. Photosynthetic genes may have additional functions in subgenus *Monogyna*, where chlorophyllous cells are also concentrated in a thin layer of internal stem tissue [5].

### Loss of photosynthesis in *Cuscuta*

If photosynthesis is important for seed lipid production in most *Cuscuta*, then questions remain as to why a few species can survive without chlorophyll and *rbcL *[16] (*C. chilensis*; Figure 1). Reproductive biology of the lineages of *Cuscuta *that contain these species, subsections Odoratae and Grandiflorae (and possibly Acutilobae), may provide an important clue. Large corolla size (see *Cuscuta chilensis*; Figure 7) and strong fragrance characterize members of these subsections. In our experience with cultivating *C. chilensis*, it is incapable of producing selfed seed (from over 100 hand-pollinations), whereas most *Cuscuta *species readily produce massive quantities of selfed seed without assistance. Observations of various natural populations in Chile showed that pollinator visitation was frequent, with species of Lepidoptera, Hymenoptera and Diptera all moving between flowers with varying amounts of *Cuscuta *pollen on their bodies. However, seed set in these natural populations was extremely low, with only a small proportion of old flowers containing viable seed. Likewise, seeds are usually sparse or absent on herbarium specimens of species in sections Odoratae and Grandiflorae. An ability to survive on hosts year-round may explain why these species have less demand for a massive seed set and, thus, are able to survive the cost of low fecundity to reap the benefits of self-incompatibility. A decreased demand for massive lipid production during fruiting may have rendered the remaining photosynthetic genes in the ancestor of these *Cuscuta *species obsolete. Our results and observations suggest in-depth molecular and reproductive physiological study of the large-flowered South American clades of *Cuscuta *subgenus *Grammica *will provide further insight into the evolutionary loss of photosynthesis in this parasitic lineage.

## Conclusion

By generating a well-supported phylogeny of the economically important parasitic plant genus *Cuscuta*, we have provided a framework through which to test whether traditional taxonomic divisions of the genus represent monophyletic evolutionary clades, to evaluate which morphological characters are systematically misleading, to formulate biogeographical hypotheses that best explain current distributions of major clades and to interpret molecular phenomenon such as nuclear genome size evolution and plastid genome evolution. Subgenus *Cuscuta *is paraphyletic with subgenus *Grammica *nested within it. Subgenus *Grammica *likely colonized the new world through a dispersal event from South Africa to South America and then radiated throughout both North and South America; subsequent long-distance dispersal events, many possibly aided by transition to floating indehiscent capsules, best explain the few scattered members of subgenus *Grammica *in Hawaii, Australia, Asia and Africa. Nuclear genome size is highly variable in the genus and may be useful in recognizing new cryptic species. A reduction in plastid genome size appears to have occurred in punctuated steps followed by periods of relative stasis. Although plastid nucleotide substitution rates are quite rapid, photosynthetic genes are very strongly conserved in the majority of *Cuscuta *species even after the loss of all plastid *ndh *and RNA polymerase genes. The plastid genome is likely retained primarily for lipid biosynthesis during seed production and is possibly lost completely in a single clade of outcrossing species whose life histories seem to accommodate a reduction in overall seed production.

## Methods

### Plant material

The quality of available tissue for different *Cuscuta *species was variable, but a common method using a typical plant CTAB DNA isolation [40] with 1% polyethylene glycol (molecular weight 8,000) added to the buffer proved effective for live plants grown in the Pennsylvania State University Biology greenhouse, freshly collected wild plants, frozen tissue, silica-gel dried tissue and small samples from herbarium specimens. For some dried material received in silica gel, vouchers were unavailable and we instead identified the species by dissection of rehydrated flowers from the sample. Photographs taken through a dissecting scope of characters necessary for identification are available as vouchers for such species. For two species for which we received no voucher material or flowering and fruiting material for dissection, we verified proper identification of the sample with sequence comparison of vouchered data at loci always variable above the species level. Vouchered specimens were deposited in the Pennsylvania State University Herbarium (PAC). Vouchers, taxon information and GenBank accession numbers for all sequences are presented in Table 3.

**Table 3:** Voucher information and GenBank accession numbers

Species	Voucher	*rbcL*	*rps2*	*matK*	ITS	*atpE*	*rpoA*
*Cuscuta cuspidata*	N/A	N/A	N/A	N/A	AF323744	N/A	N/A
*C. gronovii*	(PAC) JRM03.1206	EU330262	EU330242	N/A	EU330289	N/A	N/A
*C. gronovii *var.*calyptrata*	(PAC) JRM03.1102	N/A	N/A	N/A	EU330290	N/A	N/A
*C. rostrata*	(PAC) JRM03.1001	EU330263	EU330243	N/A	EU330291	EU330231	N/A
*C. cephalanthi*	(PAC) JRM03.1002	EU330264	EU330244	N/A	EU330292	N/A	N/A
*C. glomerata*	(TEX) 00393912	EU330265	EU330245	N/A	EU330293	N/A	N/A
*C. compacta*	(PAC) JRM03.1104	EU330266	EU330246	N/A	EU330294	N/A	N/A
*C. denticulata*	(PAC) CWD98.301	N/A	N/A	N/A	EU330295	N/A	N/A
*C. veatchii*	(PAC) JRM04.0701	EU330267	EU330247	N/A	EU330296	N/A	N/A
*C. campestris*	(PAC) JRM04.0702	EU330268	EU330248	N/A	EU330297	N/A	N/A
*C. polygonorum*	(PAC) JRM03.1207	N/A	N/A	N/A	EU330298	N/A	N/A
*C. obtusiflora*	(PAC) JRM03.0207	NC009949	NC009949	N/A	EU330299	NC009949	N/A
*C. salina*	(TEX) Halse4961	EU330269	N/A	N/A	EU330300	N/A	N/A
*C. salina *var.*apoda*	(TEX) Tiehm13405	N/A	N/A	N/A	EU330301	N/A	N/A
*C. suksdorfii*	*	N/A	N/A	N/A	EU330302	N/A	N/A
*C. subinclusa*	(TEX) provance2138		N/A	N/A	EU330303	N/A	N/A
*C. californica*	(TEX) van der Werff 1	N/A	N/A	N/A	EU330304	N/A	N/A
*C. tasmanica*	*	EU330271	N/A	N/A	EU330305	N/A	N/A
*C. tinctoria*	(TEX) 00155775	N/A	N/A	N/A	EU330306	N/A	N/A
*C. applanata*	*	EU330272	EU330249	N/A	EU330307	N/A	N/A
*C. potosina*	(TEX) 00155818	N/A	N/A	N/A	EU330308	N/A	N/A
*C. sandwichiana*	(BISH) 2098	N/A	N/A	N/A	EU330309	N/A	N/A
*C. americana*	*	EU330273	N/A	N/A	EU330310	N/A	N/A
*C. attenuata*	N/A	N/A	N/A	N/A	AF348405	N/A	N/A
*C. indecora*	(PAC) JRM03.1103	EU330274	EU330250	N/A	EU330311	EU330232	N/A
*C. coryli*	(PAC) 62115	N/A	N/A	N/A	EU330312	N/A	N/A
*C. leptantha*	(TEX) 00394072	N/A	N/A	N/A	EU330313	N/A	N/A
*C. umbellata*	*	N/A	EU330251	N/A	EU330314	N/A	N/A
*C. foetida*	(TEX) Sparre16952	N/A	N/A	N/A	EU330315	N/A	N/A
*C. chilensis*	(PAC) JRM03.0203	N/A	N/A	N/A	EU330316	N/A	N/A
*C. nitida*	*	EU330275	EU330252	EU330280	EU330317	EU330233	EU330236
*C. epilinum*	(PAC) JRM03.1210a	EU330276	EU330253	EU330281	EU330318	EU330234	EU330237
*C. europaea*	(PAC) JRM03.1101	EU330277	EU330254	EU330282	EU330319	EU330235	EU330238
*C. japonica*	#	AY101061	EU330255	EU330283	EU330320	N/A	EU330239
*C. lupuliformis*	(PAC) JRM03.0808	EU330278	EU330256	EU330284	EU330321	N/A	EU330240
*C. reflexa*	#	X61698	EU330257	EU330285	EU330322	N/A	EU330241
*C. exaltata*	*	NC009963	NC09963	NC09963	EU330323	NC09963	NC09963
*Ipomoea purpurea*	(PAC) JRM03.1203	NC009808	NC009808	NC009808	EU330324	NC009808	NC009808
*Ipomoea quamoclit *X *coccinea*	*	N/A	N/A	N/A	EU330325	N/A	N/A
*Calystegia sepium*	(PAC) JRM97.052	AY100992	EU330258	N/A	EU330326	N/A	N/A
*Jacquemontia tamnifolia*	(MO) 00883399	EU330279	EU330259	EU330286	EU330327	N/A	N/A
*Dichondra carolinensis*	#	N/A	EU330260	EU330287	EU330328	N/A	N/A
*Dichondra occidentalis*	N/A	AY101023	N/A	N/A	N/A	N/A	N/A
*Humbertia madagascariensis*	(MO) 3854462	AY101062	EU330261	EU330288	EU330329	N/A	N/A
*Nicotiana tabacum*	N/A	NC001879	NC001879	NC001879	AJ492448	NC001879	NC001879
*Atropa belladonna*	N/A	NC004561	NC004561	NC004561	N/A	NC004561	NC004561
*Panax ginseng*	N/A	NC006290	NC006290	NC006290	N/A	NC006290	NC006290
*Spinacia oleracea*	N/A	NC002202	NC002202	NC002202	N/A	NC002202	NC002202

Bold = Sequences already deposited on Genbank before this study.

*Not enough material for herbarium voucher; photographs of the dissected flowers used for identification are available upon request.

^#^No voucher available; verified by sequence identity to existing sequences on GenBank.

### PCR and sequencing

Previously designed primers ITS4 and ITS5 were used for amplification and sequencing of the nuclear ITS locus according to a published protocol [41]. A few taxa exhibited sequence polymorphisms, particularly in a highly variable loop region [42] , which was not confidently alignable across all taxa and was excluded for analyses. This also often resulted in length polymorphisms that required Topo cloning (Invitrogen, Carlsbad, CA) for capillary sequencing. For all taxa with polymorphic ITS loci, we found no evidence of lineage sorting, as all alleles from a given species always formed a clear clade. We used consensus sequences from multiple clone reads to sort true nucleotide polymorphisms from *Taq *polymerase error in incorporated PCR fragments. True nucleotide polymorphisms were rare and were entered into the data matrix as the predominant locus in our sample. Only one sequence from each species with identified length polymorphisms was used. Plastid *rps2 *was amplified with primers rps2-661R and either rps2-18F or rps2-47F [34] or, for recalcitrant taxa, new primers designed from the more readily generated *Cuscuta *sequences and the available plastid genome sequences of *C. exaltata *and *C. obtusiflora *(data analysis in prep). A partial *rbcL *product was also amplified using published primer sequences [43] or new primers designed specifically for *Cuscuta*. For some taxa sampled from herbarium material, internal primer combinations were used to amplify and sequence the gene in parts when necessary. Amplification across *atpE *was performed using primers atpB-1277F [44] and trnF-F [45]; for members of section Eucuscuta, trnT(2)-R [46] was substituted for trnF-F on the basis of an inversion of those taxa verified by this PCR and a PCR from trnF-F to rps4-32F [47]. *rpoA *or *rpoA *pseudogenes were amplified and sequenced with a combination of the newly designed primers petD-endF and rps11-C398F. PCR protocol for *rps2*, *rbcL*, *atpE*, and *rpoA *all followed the *rps2 *protocol described by dePamphilis *et al*. [34]. Long PCR assays of intergenic sequences were conducted using the following primer combinations: psbD-40F [48] to trnfM-R [46]; trnC-F [46] to psbD-45R [48]; and rps4-32F to atpB-s1277F. PCR from psbA-984F to ndhB-13F [48] was used to confirm contraction of the inverted repeat in members of subgenus *Monogyna*. These longer PCR assays were performed using 1 × *Taq *Extender Buffer, 0.2 mM of each dNTP, 2.5 mM MgCl_2_, 3.0 μM of each primer, 0.5 units of *Taq *DNA Polymerase (Promega, Pittsburgh, PA), 0.5 units of *Taq *Extender (Stratagene, La Jolla, CA) and approximately 500 ng of template DNA in 50 μl total volume. Amplification was accomplished using a thermal-cycling scheme of an initial 94°C denaturation for 2 min, followed by 10 cycles of 94°C for 10 s, 55°C for 30 s and 68°C for 6 min. Sixteen additional cycles were performed under the parameters of 94°C for 20 s, 55°C for 30 s and 68°C for 6 min with an additional 20 s added to this extension time each cycle. A final, additional extension at 68°C for 7 min was also performed. In cases where multiple bands were produced, this process was repeated with the extra MgCl_2 _removed. All newly designed PCR primers are given in Table 4. All PCR products that were sequenced were cleaned using a Qiaquick PCR Purification Kit (Qiagen, Valencia, CA) or a combination of five units of Exonuclease I and five units of Shrimp Alkaline Phosphatase (USB, Cleveland, OH) in 10 μl volume incubated at 37°C for 1 h followed by 15 min at 80°C to inactivate the enzymes. Sequencing was performed on a Beckman-Coulter CEQ-8000XL machine following the manufacturer's protocol.

**Table 4:** New primer sequences designed for this study

Primer name	Sequence (5' to 3')
*rbcL-Z1Cus*	ATGTCACCACAAACAGARACTAAARC
*rbcL-521F*	CTATTAAACCWAAATTGGGKTTATC
*rbcL-599R*	GTAAAATCAAGTCCACCRCGAAG
*rbcL-818F*	GATTCACTGCAAATACTTCTTTGG
*rbcL-910R*	GTCTATCAATAACKGCATGCATTG
*rbcL-1392R*	CTCYTTCCATACCTCACAAGCAG
*rps2-J12F*	ATATTGGAACATMAAWTTGGAAG
*rps2-J662R*	CYAATTTGTTMAGAATGAATCG
*rps2-J306F*	CGGTATGTTAACRAATTGGTCCAC
*rps2-J458R*	CCCAGATATMTTTGCAAGCGAGC
*petD-endF*	CAAAATCCATTTCGKCGTCCAG
*rps11-C398F*	GCCACACAATGGCTGTAGACCTCC

### Phylogenetic analyses

ITS sequences were initially aligned using Clustal X [49] followed by manual adjustment. Protein-coding plastid sequences were easily aligned by eye, with attention paid to codon alignment in the few areas where gaps existed. A consensus of 500 bootstrap trees was created for each gene individually using maximum parsimony in PAUP*4.0b10 [50]. Aligned datasets contained 684 base pairs (bp) for ITS, 1,399 bp for *rbcL*, and 660 bp for *rps2*. A combined bootstrap consensus was created using data from these three genes combined with *matK *data (1,650 aligned bp, 4,393 combined bp) [51] , although not all taxa are available for every locus owing to gene loss and/or failed amplification. Bayesian posterior probabilities were calculated for each node using Mr. Bayes v3.0b4 [52]. Four cold chains and one chain heated at the default value were run with swapping according to default settings and a general-time reversible (GTR) likelihood model with a gamma and invariant parameter estimated from the data. One million generations were run with sampling every hundredth generation for a total of 10,000 trees. Likelihood estimates were graphed to determine appropriate burn-in values for each gene (200 trees discarded for *rps2 *and *rbcL*, 400 trees discarded for ITS, 250 discarded for combined data). In addition, maximum-likelihood phylograms and non-parametric bootstrap values (100 replicates) were generated with the program Garli (Version 0.951) using default search options under the GTR + gamma + I model for each of the three newly reported gene alignments with parameters estimated from the data.

### Genome size estimates

Nuclear genome size estimates and standard errors were measured by flow cytometry [53] using either rice, soybean, tobacco, barley or wheat cultivars of known nuclear genome size as standards. Four replicates were performed for each plant, with the mean estimates and standard deviations (SD) reported in Table 1. Fresh plant material for these measurements was grown in the Pennsylvania State University Biology greenhouse. *Cuscuta *seeds were germinated after scarification in concentrated H_2_SO_4 _and grown with *Impatiens walleriana*, *Solenostemon scutellarioides *or *Linum usitatissimum *(for *C. epilinum*) as hosts. Fresh stem tip tissue was used for all size estimates reported.

### Rates analyses

Aligned datasets for *atpE*, *rbcL *and *rps2 *with identical sampling of 12 taxa were imported into HYPHY.99beta (see [54]). A different set of taxa was used for *rpoA*, which is missing in all sampled members of subgenus *Grammica*. A user tree, based on highly supported nodes of the bootstrap consensus tree in Figure 2 that was congruent with all single-gene analyses, was used for all genes (single-gene trees for *atpE *and *rpoA *not shown). Synonymous and non-synonymous branch lengths were first calculated with no constraints under the MG96, HKY 3, 4 codon model. Next, a tree with all branches constrained to the same non-synonymous to synonymous ratio was optimized, and a likelihood ratio test (LRT) was performed to determine whether the unconstrained tree had a significantly better likelihood. Likelihood parameters were then reoptimized for trees with the non-synonymous to synonymous rate ratio constrained differently for various clades (i.e. two non-synonymous to synonymous rate ratios on the tree; one for the subclade being tested, one for the remainder of the tree). Clades examined in this manner for *atpE*, *rbcL *and *rps2 *were the Convolvulaceae clade (*Ipomoea *+ *Cuscuta*), all *Cuscuta*, all *Cuscuta *except subgenus *Monogyna *and the clade comprising the three sampled species of subgenus *Grammica*. For *rpoA*, clades examined were Convolvulaceae, *Cuscuta*, subgenus *Cuscuta *and *Cuscuta nitida*. LRTs were confined to testing only hypotheses of change at these nodes of interest rather than performing numerous additional tests and thereby increasing the chance of Type I error.

## Authors' contributions

JRM collected PCR data, performed analyses and wrote the manuscript. KA performed flow cytometry nuclear genome size estimation. JVK and JLB helped produce complete plastid genome sequences for two *Cuscuta *species and a photosynthetic outgroup from which some loci were extracted for this study and which were used extensively for primer design. CWD participated in design and coordination of the research and extensively edited the manuscript. All authors read and approved the final manuscript.
